# Hypnotism in Therapeutics

**Published:** 1888-05

**Authors:** 


### HYPNOTISM IN THERAPEUTICS.

*Dr. Moll* states that he has seen at least one thousand single experiments, which enables him to affirm that a large part of all that has been written about hypnotism, howsoever unlikely it may sound, is perfectly true. According to *Senator*, we have to view hypnotism as a condition in which an individual is deprived of his own will and becomes the passive instrument of the manipulator. *Braid*, of Manchester, has published, in 1843, the first treatise on hypnotismus, trying at the same time to turn it to account as a therapeutic agent. He also discovered *suggestion* and described it. "Suggestion" means any physical action on the hypnotized subject. *Moll* exemplifies this in the following manner: A subject is just speaking ; the experimenter calls out to him : "You can't speak any more, you have lost your language !" The subject continues to perform some motions for the purpose of speaking, but is unable to do so.

Recently, thorough investigations have been commenced in France, particularly in the Salpetriere of Paris, under *Charcot's* direction. They distinguish a major and a minor hppnosis ; the former alone is typical and is met with in hysteria. Three stages of hypnotism mav be distinguished :

1. *Lethargy.* It may be produced by causing the subject to fix his looks on some object not to much resplendent. The lethargic individual seems to be asleep ; his lids are closed ; on raising them, they drop back without power. In this state there exists a hyperecitability of the nerves and muscles.

2. *Catalepsy.* For this condition, an individual can be given any arbitrary position in which he continues wthout change. The eyes are open, the look is staring. The individual can be transferred from catalepsy to lethargy by closing his eyes.

3. *Somnambulism.* The individual has his eyes wholly or half closed, and resembles a somnambulist. There is no excessive excitability of muscles, but by slight irritations of the skin the subjacent muscles may be subjected to contraction. This state can be produced by gentle friction or pressure on the vertex.

*Moll* himself has succeeded in curing, by means of hypnosis, neuralgias in patients who had resisted prolonged electric treatment, embrocations, etc., and in restoring some neurasthenics to psychical repose ; also in rapidly freeing from headache, persons who had not received any advantage from antifebrine nor from antipyrine.

Yet, hypnotic treatment has also its inconveniences. It is not possible to hypnotize all individuals, nor, in case of success, to have the hypnotic condition always in sufficient intensity, or there is no improvement in spite of hypnosis ; the latter result is not an infrequent one. Oftimes, hypnotization is followed by a condition of weariness, but this condition can soon be overcome. It is self-evident that all psychical emotions, as in public exhibitions were frequently met with, have to be avoided.

The time has not yet arrived to clearly define the indications for hypnotic treatment ; taking into account the literature of the French, this method seems to be applicable to hysteria, neuralgia, psychical conceptions, insomnia, etc. It is stated that hypnotism has alreedy been successfully applied in morphiophagy, alcoholism, enuresis, dysmenorrhœa, etc. In a ticular rheumatism, pain has been greatly appeased, before any medicament was given. Whether the fundamental disease will be modified in this way, is, of course, another question ; yet the possibility of organic modifications cannot be excluded ; for any part of the body, subjected to concentrated attention, will alter its conditions of alimentation. *Braid* affirms having succeeded in healing an abscess of the cornea by means of hypnosis, and some modern publications clearly demonstrate how large an influence suggestion may exercise on the organism. In one individual the following result was obtained : On informing him that at a certain time a certain determined place of his arm would be subjected to bleeding, it was observed that this place first commenced reddening, then showing in relief a letter determined beforehand, and finally some drops of blood being squeezed out. The miricle of Louise Lateau might thus be explained quite naturally. In another instance, some postal stamps were pasted on the back of an individual who was told that it was a blister plaster. There was indeed a blister forming on this spot, and in another case a simple hyperæmia. On the other hand blister-plasters was spread out on cigarette paper, and it was applied with the statement it was linen paper ; in this case, no blister, it is asserted, was produced. A piece of cold iron was placed in the hand of another person, suggesting that it was glowing ; the hand showed blisters from burning. *Moll* corroborates from his own experience the fact that some persons, by the suggestion of taking ammonia, are being made to sneeze ; and other persons, by the suggesfion of taking alcohol or champagne, are being subjected to a condition like intoxication. Yet, it seems that a certain hypnotic education is necessary for all the observations and experiments.

On the other hand, mere negation is no longer admissable, although until now it was not possible to give a self explanation of the successes of hypnotic treatment. There is certainly no panacea in hypnotic treatment, but the results, reported from France, are altogether highly encouraging. *Voisin*, in the Paris Salpetriere, has obtained good results in cases of interrupted menstruation by simple suggestion that menses would appear on such or such a determined day. Yet, the actual value of hypnotism as a therapeutic agent will not be established by personal impressions, it will have to be measured by the nnmber of failures which each experimenter will meet with. Future impartial observation will be required to settle this question of the therapeutic value of hypnotic treatment.—*Abstract from Deutsche Medizinal Zeitung, Berlin.*

